# Sample preparation method for metal(loid) contaminant quantitation in rodent hair collected in Yuma County, Arizona

**DOI:** 10.1007/s10661-021-09292-8

**Published:** 2021-07-27

**Authors:** Jonathan Credo, Amy Chandos, Camilla Checinski, Frank A. von Hippel, Jani C. Ingram

**Affiliations:** 1 College of Medicine, University of Arizona, Tucson, AZ USA; 2 Department of Biological Sciences, Northern Arizona University, Flagstaff, AZ USA; 3 Department of Chemistry & Biochemistry, Northern Arizona University, Flagstaff, AZ USA; 4 Community, Environment and Policy, Mel and Enid Zuckerman College of Public Health , University of Arizona, Tucson, AZ USA

**Keywords:** ICP-MS, CV-AAS, Agrichemical exposure, Sample preparation, Rodent animal model

## Abstract

Yuma County, Arizona, is a large agricultural hub of the USA located in the southwestern corner of Arizona on the USA-Mexico border. Year-round use of agrichemicals at a massive scale along with the influx of aquatic contaminants in the Colorado River led to significant levels of environmental pollution and hence exposure risks for people and wildlife. Although hair is a recognized biomarker for metal exposure, there is no universal hair preparation protocol. This study evaluated two digestion methods for metal quantitation using inductively coupled plasma-mass spectrometry (ICP-MS) and three methods for mercury quantitation using cold vapor-atomic absorption spectroscopy (CV-AAS), both employing certified reference materials. The “overnight” and “heating” digestion methods were suitable for ICP-MS, while only the heating method was suitable for CV-AAS. These validated methods will be useful for a variety of human and wildlife assessments of toxic metal(loid) exposure.

## Introduction

Yuma County, Arizona, is an important agricultural region of the USA as it provides more than 90% of leafy greens consumed in the USA during winter, in addition to many other crops (Kerna & Frisvold, 2014). To maintain high agricultural yields, more than 2.6 million kilograms of organic and inorganic based pesticides is applied to fields each year (Sugeng et al., 2013). Pesticide exposure represents a significant health concern for humans and wildlife due to the heavy use in Yuma County, application techniques, and wind patterns that mobilize pesticides across the region. A myriad of detrimental health effects is linked to pesticide exposure, which depend on the length and dose of the exposure along with the type of pesticide (Alavanja et al., 2004; Baldi et al., 2011; Lizardi et al., 2008). Lizardi et al. (2008) evaluated the neurobehavioral performance of Hispanic children from Yuma County and other southern Arizona agricultural communities and demonstrated that exposure to organophosphate pesticides led to deleterious mental functioning and performance. To date, studies conducted in Yuma County have focused on organic pesticide exposure, leaving a knowledge gap on the effects of inorganic pesticides on the population and environment (Lizardi et al., 2008; Sugeng et al., 2013).

In this paper, we evaluate three digestion protocols for the quantitation of toxic metal(loid)s in rodent hair to validate these methods for studies of human and wildlife health effects due to exposure. Several toxic metal(loid)s are active ingredients of historically used inorganic pesticides (lead (Pb), mercury (Hg), arsenic (As)), while others are still used in agriculture (manganese (Mn), copper (Cu)) (Sugeng et al., 2013). Furthermore, many other sources of exposure to toxic metal(loid)s exist in Yuma County and throughout the world. Hair was chosen because it represents a non-invasive and easy-to-gather biomarker for exposure that has been used to assess body burden (Gellein et al., 2008; Pirrone et al., 2013). Animal keratin, including rodent hair, is frequently used to assess exposure burden (Appenzeller et al., 2017; Beernaert et al., 2007; Jaspers et al., 2019; Lettoof et al., 2021). Unfortunately, some metals used in inorganic pesticides have not been sufficiently studied in hair, and there is a lack of consensus on hair preparation and digestion methods (Ishak et al., 2015; Pozebon et al., 2017). This is exemplified by the meta-analysis conducted by Pozebon et al. (2017), which reviewed the current literature for hair preparation and digestion protocols. Protocols were evaluated using certified reference materials (CRMs) and selected based upon feasibility given small sample masses of hair. The goal is to determine the most efficacious method for analysis of toxic metal(loid)s in hair samples from rodents collected in Yuma County, and more generally for similar studies elsewhere.

## Materials and methods

### Rodent sample collection

**Fig. 1:**
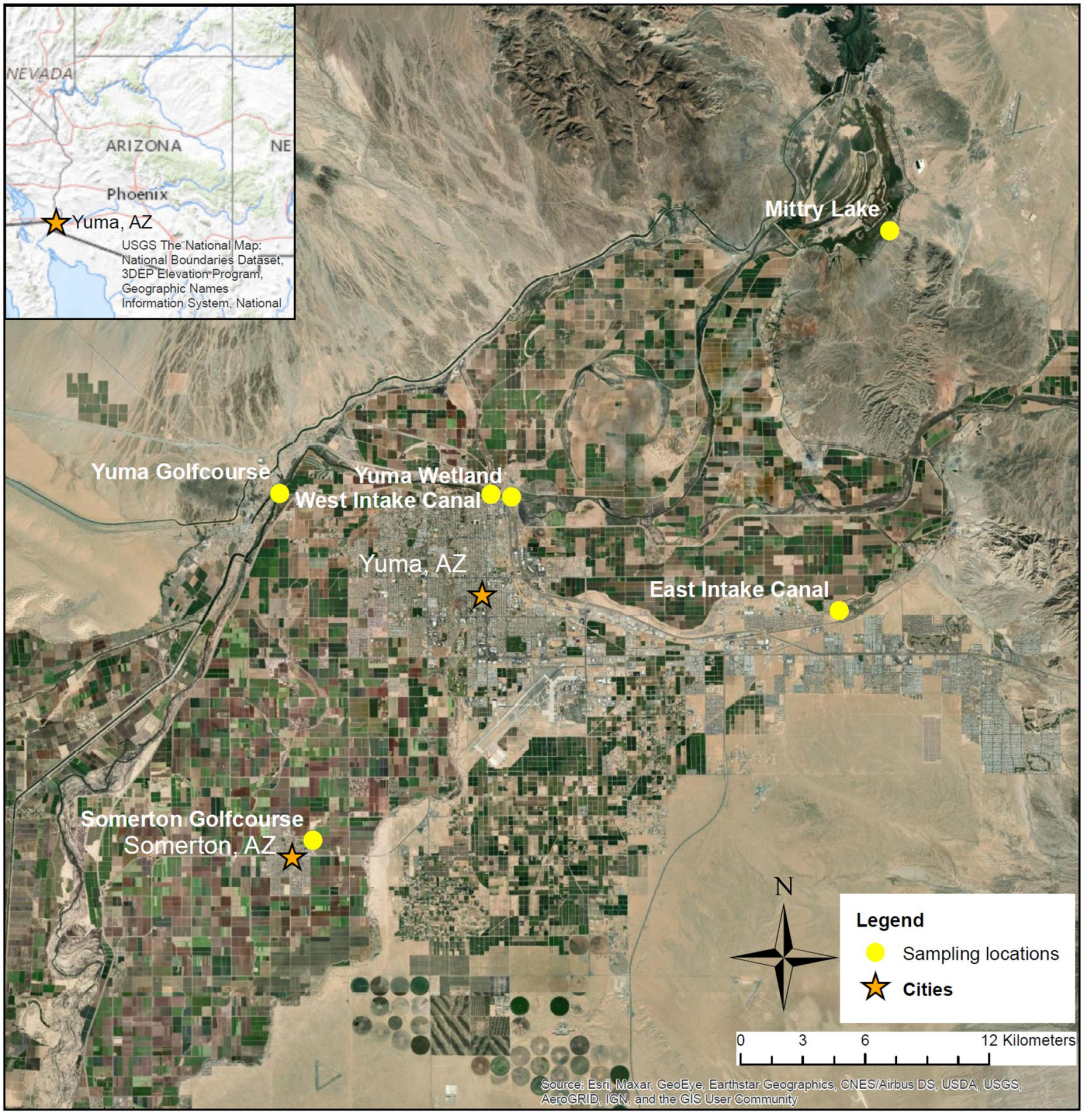
Study region in Yuma County, Arizona. Rodent collection sites are denoted by yellow circles. Map insert shows location of Yuma County, AZ

Rodents were collected using Sherman live traps, baited with peanut butter and birdseed, at seven sites across Yuma County, from November 2018 through November 2019. Rodents were captured in riparian areas adjacent to agricultural lands and the Colorado River within the municipalities of Yuma and Somerton, as well as at our reference site at Mittry Lake, a natural wetlands area fed by the Colorado River located 20 km north of Yuma and 15 km upstream from the nearest agriculture (Fig. 1). Traps were placed at sampling locations at dusk, and then checked the following morning at dawn. Male and female adult rodents were kept, and juveniles were released. Captured rodents were either transported to the lab at the NAU Yuma campus or field dissected. All rodents were humanely euthanized following established American Veterinary Medical Association procedures using isoflurane (Underwood & Anthony, 2020). Morphological characteristics (e.g., species identification, body length, body weight, sex) were recorded prior to hair being collected from their dorsum. Hair was shaved with a trimmer, which was sterilized between animals. Additional tissue samples were collected (e.g., blood, gonads, thyroid) for separate histological, endocrinological, and gene expression assays to be reported elsewhere. Hair was placed in envelopes, given an alphanumeric code, and transported to NAU Flagstaff for preparation and analysis. Of the species captured, only *Peromyscus eremicus* (cactus mouse) hair was used in the current study because they were the most abundant. Rodent collection was approved by the Northern Arizona University (NAU) Institutional Animal Care and Use Committee (protocol 17–013) and the Arizona Department of Game and Fish (Licenses SP611340 & SP639726).

### Instrumentation and method validation

All analyses were conducted at the NAU Flagstaff campus. Total Hg was quantified using a Perkin-Elmer FIMS 100 CV-AAS with a PerkinElmer S10 Autosampler, and elemental analysis (As, cadmium (Cd), Cu, and Pb) was accomplished using a Thermo X Series II ICP-MS with standard glass spray chamber introduction. Standard operating parameters were used for the FIMS 100 (EPA, 2007a; McIntosh, 1993). Prior to each analysis, the ICP-MS was tuned using Tune Solution A (Inorganic Ventures). ERM-DB001 Human hair (trace elements, from the European Commission Joint Research Centre) and NIST 1640a (trace elements in natural water, from the National Institute of Standards and Technology) CRMs were used to assess digestion and instrument efficiency. Rhodium (Rh) and iridium (Ir) at 1.00 μg/L were used as internal standards for ICP-MS analysis.

### Digestion methodology

**Fig. 2:**
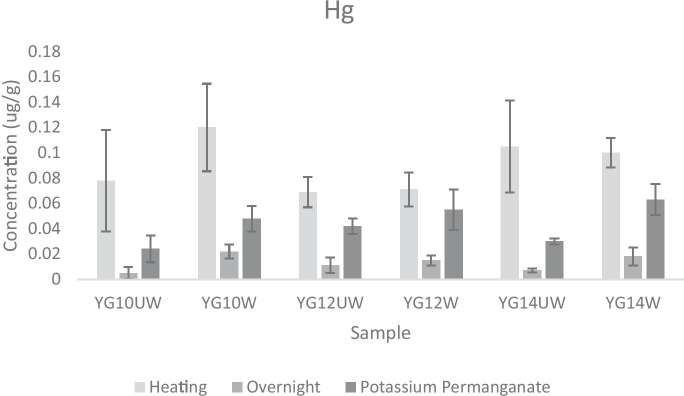
Comparison of the effect of washing rodent hair samples on Hg concentration as evaluated by CV-AAS. Samples labeled “UW” represent unwashed hair samples and samples labeled “W” represent washed hair samples. The legend at the bottom denotes the different digestion method (left to right—“heating,” “overnight,” “potassium permanganate”). Standard deviation is represented as capped bars for each sample

Three digestion methods were adapted from previously published protocols and performed using hair from one of three rodents (YG10, YG12, YG14): (1) heating, (2) overnight, and (3) potassium permanganate (KMnO_4_; Table 1) (Ishak et al., 2015; Pozebon et al., 2017). For each method, the digestion efficiency was evaluated with a CRM (ERM-DB001 Human Hair, washed and powdered) (Astolfi et al., 2020; Cabral Pinto et al., 2019). All three digestion methods were evaluated for total Hg quantitation (Fig. 2). For the heating method, hair samples (30–40 mg) were digested with 1400 μL trace metal clean concentrated hydrochloric acid (HCl; 35%; Fisher Chemical) and 75 μL suprapur hydrogen peroxide (H_2_O_2_; 30%; Sigma-Aldrich), and placed in a 100 °C isotherm for 90 min. Prior to analysis, samples were diluted to 15 mL with nanopure water (NP) and filtered with Whatman GD/X PVDF 0.45-μm syringe filters. For the overnight method, the same protocol for heating was followed with the addition of a 12 h overnight digest (also in 1,400 μL HCl and 75 μL H_2_O_2_) prior to samples being placed in the isotherm. For the potassium permanganate method, samples of hair (500 mg) were digested with 5 mL HCl, 2 mL 5% (w/v) KMnO_4_ (Acros Chemical), and 30 mL of distilled water. Samples were digested for 24 h and then placed into an 80 °C water bath for 2 h. After digestion, samples were diluted to a final volume of 50 mL, filtered (VWR qualitative 33 cm filter paper), and the KMnO_4_ was neutralized with 10% hydroxylamine (Fisher Chemical) and two–three drops of XIAMETER AFE-0110 Antifoam Emusion added (Dow Chemical).

**Table 1:** Summary of the three hair digestion methods used for Hg analysis by a Perkin-Elmer FIMS 100 CV-AAS

Protocol name	Sample weight (mg)	Chemical digest	Heating	Dilution and filtration
Heating	30–40	1400 μL HCl + 75 μL H_2_O_2_	90 min 100 ºC isotherm	Diluted to 15 mL with NP, filtered
Overnight	30–40	1400 μL HCl + 75 μL H_2_O_2_	12 h overnight digest + 90 min 100 ºC isotherm	Diluted to 15 mL with NP, filtered
Potassium Permanganate	500	5 mL HCl, 2 mL 5% (w/v) KMnO_4_, 30 mL distilled water	24 h digest + 120 min 80 ºC water bath	Diluted to 50 mL with distilled water, filtered, 5 drops 10% hydroxylamine and antifoaming agent

**Fig. 3:**
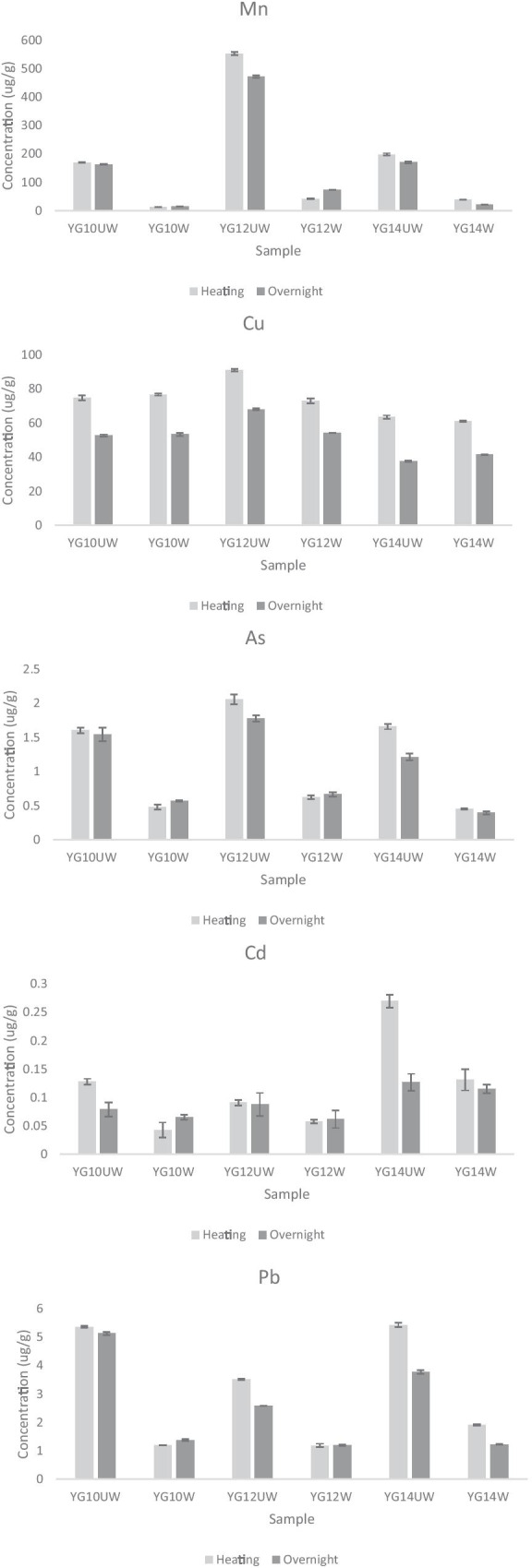
Comparison of effect of hair washing on rodent hair samples as evaluated by ICP-MS. Both the heating and overnight digestion methods were compared again, because both had comparable recoveries for ICP-MS analysis on washed samples. Samples labeled “UW” represent unwashed hair samples and samples labeled “W” represent washed hair samples. The legend at the bottom denotes the different digestion method (left is heating and right is overnight). Standard deviation is represented as capped bars for each sample

The heating and overnight digestion methods were also evaluated by ICP-MS and utilized the same rodent hair samples (YG10, YG12, YG14) (Table 2; Fig. 3). For the heating method, samples of hair (20 mg and 200 mg) were digested with 1500 μL of OPTIMA grade nitric acid (Fisher Chemical) and 80 μL H_2_O_2_, and placed into a 100 °C isotherm for 90 min. After digestion, samples were diluted to 15 mL with NP and filtered. For the overnight method, the same protocol for heating was followed with the addition of a 12 h overnight digest prior to samples being placed in the isotherm.

**Table 2:** Summary of the two hair digestion methods used for elemental analysis by a Thermo-Fisher X-Series II ICP-MS

Protocol name	Sample weight (mg)	Chemical digest	Heating	Dilution and filtration
Heating	30–40 & 200	1500 μL HNO_3_ + 80 μL H_2_O_2_	90 min 100 ºC isotherm	Diluted to 15 mL with NP, filtered
Overnight	30–40 & 200	1500 μL HNO_3_ + 80 μL H_2_O_2_	12 h overnight digest + 90 min 100 ºC isotherm	Diluted to 15 mL with NP, filtered

### Hair washing

The impact of hair washing was evaluated on three rodent samples using a modified International Atomic Energy Agency protocol (Pozebon et al., 2017). Hair samples were washed with acetone (CHROMASOLV for HPLC > 99.8%, Sigma Aldrich), rinsed three times with NP water, and rinsed with acetone again, and then 20–40 mg of washed and unwashed sample was digested by the methods above and analyzed by ICP-MS and CV-AAS.

## Results and discussion

Comparison of the three digestion methods for CV-AAS showed that all three methods resulted in Hg values lower than the expected value based on the CRM (Table 1). This is expected due to the high volatility of Hg. Of the three methods, the heating method resulted in the only accuracy that fell within acceptable recovery limits (USEPA, 2007). This is likely due to the reduced time for the digest leading to less Hg loss due to volatilization (USEPA, 2007).

We compared the efficacy of digestion methods used on hair samples for ICP-MS (As, Cd, Cu, and Pb). The concentration of each analyte represents an average of three replicates and is reported in μg/g (ppm; Tables 3 and 4). Additionally, the tables present the nominal concentrations for the CRM. Concentrations of the samples were compared to the CRM with the lower and upper uncertainties and are represented as a percent error range. Comparison of the digestion methods to the CRM demonstrated that the “overnight 20 mg” and “heating 20 mg” methods had better accuracy in the ICP-MS analysis than did the “overnight 200 mg” and “heating 200 mg” methods. The decrease in percent recovery for both the overnight 200 mg and heating 200 mg methods is likely attributed to incomplete digestion and would require an increase in the volume of HNO_3_ and H_2_O_2_ used. The primary difference between the overnight 20 mg and heating 20 mg methods was the increased recovery of Pb from the heating method, which may be due to the concentration of Pb being near the detection limit of the instrument and therefore increased instability.

**Table 3:** Comparison of the two digestion methods, each with two approximate masses of CRM used, evaluated by ICP-MS. The elements and isotopes compared are displayed at the top of table. The second row shows the true reported value and uncertainty, 95% confidence with coverage factor *k* = 2, of the CRM in μg/g. This is followed by the digestion method average reported value in μg/g. The final rows show percent error based on the reported value compared to the true value

	Element	65 Cu	75 As	111 Cd	206 Pb	207 Pb	208 Pb
	True (μg/g) (uncertainty)	33 (4)	0.044 (0.006)	0.125 (0.007)	2.14 (0.20)	2.14 (0.20)	2.14 (0.20)
Method average (μg/g)	Overnight 20 mg	33.481	0.036	0.105	2.045	2.133	2.091
	Overnight 200 mg	20.800	0.025	0.063	1.554	1.624	1.597
	Heating 20 mg	31.602	0.038	0.105	3.885	4.067	3.991
	Heating 200 mg	18.929	0.023	0.066	1.577	1.652	1.624
Method percent error (%)	Overnight 20 mg	9.51–15.45	4.08–27.10	11.23–20.65	5.41–12.61	8.86–9.93	7.76–10.66
	Overnight 200 mg	28.28–43.78	35.27–50.81	46.33–52.02	19.89–33.58	16.31–30.62	17.68–31.75
	Heating 20 mg	8.97–14.59	0.40–23.69	11.22–20.64	66.02–100.25	73.78–109.62	70.55–105.72
	Heating 200 mg	34.73–48.84	39.00–53.64	43.67–49.64	18.71–32.60	14.86–29.41	16.28–30.59

**Table 4:** Comparison of the three Hg digestion methods evaluated by CV-AAS. Average reported value in μg/g is displayed at the top, followed by the true value and uncertainty, 95% confidence with coverage factor *k* = 2, of the CRM. The final row is a percent error calculated by comparing the average reported value to the true value of the CRM

Method	Heating	Overnight	Potassium permanganate
True (μg/g) (uncertainty)	0.365 (0.028)	0.365 (0.028)	0.365 (0.028)
Average (μg/g)	0.294	0.082	0.097
Percent Error (%)	12.9–25.3	75.8–79.3	71.2–75.3

Except for testing different sample masses, all digestion methods were repeated in the washing experiment. Additionally, Mn was added to the ICP-MS analysis as it is a contaminant of concern for our study in Yuma and many other studies elsewhere, and exposure often occurs through dust inhalation (Amir Abdul Nasir et al., 2018). Mn was not included in the initial digestion comparison experiment because it is not an analyte quantitated in the CRM. As all rodents in the study are desert animals that burrow in the soil, we expected that washing the hair would remove external contaminants. Further, the hair CRM used was prepared from washed human hair samples. Therefore, washing samples would represent a closer match between sample preparation and the CRM. We found that washing resulted in a loss in analyte concentration, except for Cu, Cd, and Hg (Figs. 2 and 3), revealed by ICP-MS and CV-AAS regardless of the digestion method followed. It is possible that these metals are not major components of the soil or that rodents had metabolically deposited these metals in their hair but were not subjected to appreciable concentrations from dust.

A comparison of overnight and heating digestion method ICP-MS results revealed that for some analytes, overnight values were lower than values for heating (Fig. 3). This decrease in analyte concentration was not consistent across different analytes or even within the same sample. A possible explanation for these inconsistencies is the amount of concentrated H_2_O_2_ used during the digestion. H_2_O_2_ is commonly used in digestions and ICP-MS applications and is known to effect signal fidelity (Muller et al., 2016; Pappas, 2012). Between the overnight and heating approaches, there was a 12 h difference in time that could allow the H_2_O_2_ to off-gas in the overnight samples compared to the heating samples. This could decrease the impact of H_2_O_2_ on signal fidelity and, ultimately, analyte concentration.

The current methods were selected based on their simplicity and similarity to those used in prior studies of environment samples (e.g., soil and plants) (EPA, 2007b; Shin et al., 2019). Other sample preparation and digestion methods may result in higher accuracy for analyses of metal(loid)s in both human and animal hair. Methods were only evaluated with samples of rodent hair; similar experiments should evaluate the efficacy of these methods for the analysis of human hair to determine ideal approaches for human exposure and health studies. We expect such a study would reveal similar patterns to those seen in the analysis of rodent hair.

## Conclusions

Hair washing impacted analyte concentrations as revealed by ICP-MS, notably decreasing their concentrations compared to unwashed samples. This was expected because the purpose of washing is to remove exogenous contaminants. While there are other variables that may influence the concentration of analytes in rodent hair (e.g., age of animal and length of exposure), the purpose of this study was to evaluate published literature and develop a consistent protocol that can be used for a larger study. Of the methods investigated for both ICP-MS and CV-AAS, two methods proved suitable for ICP-MS and one for CV-AAS. Although both overnight 20 mg and heating 20 mg were efficacious, overnight 20 mg has benefits due to ease of sample preparation and efficiency of handling large numbers of samples. A single heating digestion on the day of analysis (heating method) proved to be the best approach for total Hg quantitation, likely due to a decrease in Hg volatilization.

## Data Availability

The datasets generated during and/or analyzed during the current study are available from the corresponding author on reasonable request.

## Code availability

Code availability is not applicable to this article as no code software were generated or analyzed during the current study.
